# Is early use of sodium-glucose cotransporter type 2 inhibitor (SGLT2i) necessary even in diabetic patients without cardiovascular disease: a prospective study regarding the effect of SGLT2i on left ventricular diastolic function

**DOI:** 10.1186/s44348-024-00043-0

**Published:** 2025-01-13

**Authors:** Kina Jeon, Shin Yi Jang, You-Bin Lee, Jihoon Kim, Darae Kim, Sung-A Chang, Sung-Ji Park, Sang-Chol Lee, Seung Woo Park, Moon-Kyu Lee, Eun Kyoung Kim, Kyu Yeon Hur

**Affiliations:** 1 Division of Cardiology, Department of Internal Medicine, Konkuk University Medical Center, Seoul, Republic of Korea; 2 Division of Cardiology, Department of Medicine, Heart Vascular Stroke Institute, Samsung Medical Center, Sungkyunkwan University School of Medicine, Seoul, Republic of Korea; 3 Division of Endocrinology and Metabolism, Department of Medicine, Samsung Medical Center, Sungkyunkwan University School of Medicine, Seoul, Republic of Korea; 4 Division of Endocrinology and Metabolism, Department of Internal Medicine, Uijeongbu Eulji Medical Center, Eulji University, Uijeongbu, Republic of Korea

**Keywords:** Sodium-glucose cotransporter 2 inhibitors, Diabetes mellitus, Cardiovascular diseases, Diastolic heart failure, Diabetic cardiomyopathies

## Abstract

**Background:**

There are insufficient studies to determine whether sodium-glucose cotransporter type 2 inhibitors (SGLT2i) will help reduce early diabetic cardiomyopathy, especially in patients without documented cardiovascular disease.

**Methods:**

We performed a single center, prospective observation study. A total of 90 patients with type 2 diabetes patients without established heart failure or atherosclerotic cardiovascular disease were enrolled. Echocardiography, cardiac enzyme, and glucose-control data were examined before and 3 months after the administration of SGLT2i (dapagliflozin 10 mg per day). Cardiovascular risk factors included hypertension, smoking, obesity, dyslipidemia, and old age. The primary end point was the change of E/e’ before and after administration of SGLT2i.

**Results:**

Most patients (86.7%) had three or more cardiovascular risk factors, and about 32% had all five risk factors. Although the decrease in E/e’ after the administration of SGLT2i was observed in 20% of enrolled patients, there was no significant difference in average E/e’ value or left atrial volume index before and after the SGLT2i medication. Even in patients with all known risk factors including old age, E/e’ value did not decrease after adding SGLT2i (8.9 ± 2.4 vs. 8.7 ± 3.2). There was a statistically significant difference in E/e’ change after the SGLT2i administration between patients younger than 60 years and those older than 60 years (–0.7 ± 2.2 vs. 1.1 ± 2.8, *P* = 0.002).

**Conclusions:**

In type 2 diabetes patients without documented cardiovascular disease including heart failure, administration of SGLT2i showed no improvement in diastolic function profile. Further large-scale randomized studies are needed to determine who will benefit from potential cardiovascular events with early addition of SGLT2i.

## Background

Diabetes is a major risk factor for both atherosclerotic cardiovascular disease (ASCVD) and heart failure (HF). Its risk is increased with accompanying comorbid factors such as age, dyslipidemia, obesity, and hypertension. Diabetic cardiomyopathy specifically refers to cardiac dysfunction without definite coronary artery disease or severe hypertension in diabetic patients. Diabetic cardiomyopathy initially presents as myocardial hypertrophy or diastolic dysfunction which frequently progresses to overt HF [1–4]. Given that diastolic dysfunction is an early finding of diabetic cardiomyopathy, there is increasing interest to improve diastolic function in order to reduce the morbidity of cardiovascular disease in diabetic patients.

Recently, sodium-glucose cotransporter type 2 inhibitor (SGLT2i) is widely used as an oral hypoglycemic agent in type 2 diabetic mellitus (T2DM) patients. Since SGLT2i not only reduces glucose level but also improves cardiovascular outcome in diabetic patients with established HF [5–9], the use of SGLT2i as initial treatment for glucose control is rapidly increasing. However, it is controversial whether SGLT2i should be selected as a primary option in diabetic patients with only HF complicating risk factors but without documented HF or even in patients without cardiovascular risk factors. While several societies recommend SGLT2i in patients who are at high cardiovascular risk, including those with established cardiovascular disease, other guidelines recommend a glucocentric approach and leave SGLT2i as alternative options [10, 11].

A few studies have reported changes in diastolic function after administration of SGLT2i, but those studies consisted of a small sample size or inhomogeneous population [12–14]. There are insufficient studies to determine whether SGLT2i will help reduce early diabetic cardiomyopathy, especially in patients without documented cardiovascular disease. The aim of this study was to investigate whether SGLT2 inhibitors improve diastolic function, which is accepted as a major mechanism for the development of diabetic heart failure in diabetes patients without documented HF or ASCVD.

## Methods

### Study design and patients

This study was designed as a single center, prospective, cross-sectional study. We included T2DM patients without HF who had been taking at least one oral hypoglycemic agent other than SGLT2i. All the patients did not have a history of HF or coronary artery disease regardless of the presence of cardiovascular risk factors. Patients with the following features were excluded from enrollment: (1) age < 20 or > 85 years old; (2) T2DM with hemoglobin A1C (HbA1c) < 6.5% or > 11.0%; (3) type 1 diabetic mellitus; (4) a history of HF or current HF at the time of enrollment; (5) atrial fibrillation; (6) more than moderate degree of valvular heart disease; (7) patients taking diuretics or steroid; (8) a history of hypersensitivity to SGLT2i; (9) severe renal dysfunction with estimated glomerular filtration rate < 30 mL/min/1.73 m^2^; (10) genetic problems such as galactose intolerance, lactase deficiency or glucose-galactose malabsorption; and (11) pregnancy.

### Study protocol and outcome

Among T2DM patients additionally treated with SGLT2i (10 mg per day of dapagliflozin) for the first time due to insufficient glycemic control, those who consented to their participation in this study underwent echocardiography before and 3 months after the SGLT2i medication to identify changes of cardiac systolic and diastolic function. HF complicating cardiovascular risk factors included hypertension, smoking, obesity, dyslipidemia, and old age (older than 60 years). The primary end point was the change of E/e’ before and after administration of SGLT2i. Additionally, we assessed cardiac biomarker including N-terminal pro-brain natriuretic peptide (NT-proBNP) and troponin T as well as parameters reflecting glucose control such as HbA1c, and HOMA-IR (homeostatic model assessment for insulin resistance) as an insulin sensitivity index from the fasting plasma glucose and insulin level. The last follow-up date was February 2022. In addition, we collected major cardiac events such as cardiac death, coronary artery disease and hospitalization due to HF from the medical record up to May 2024.

Comprehensive transthoracic echocardiography was performed with commercially available equipment (Vivid 7, GE Medical Systems; Acuson 512, Siemens Medical Solution; or Sonos 5500, Philips Medical System). Standard two-dimensional, color, and tissue Doppler imaging was performed with positional change. The left ventricular (LV) ejection fraction was assessed by biplane Simpson rule using manual tracing of digital images. The pulse-wave Doppler transmitral inflow velocity was obtained from an apical four chamber view for assessment of diastolic function in accordance with current guidelines using a combination of echocardiographic variables, mitral inflow velocity of the early phase (E) and late phase (A) during diastole, deceleration time, and pulsed-wave Doppler-derived mitral annular velocity imaging in the septal wall (e’). The presence of diastolic dysfunction was confirmed by decreased mitral annulus velocity (septal e’ < 0.08 m/sec) and enlarged left atrial volume (left atrial volume index [LAVI] > 34 mL/m^2^). The change of E/e’ as a parameter accurately reflect LV diastolic function and filling pressure were assessed.

### Statistical analysis

Baseline demographic data and clinical variables were summarized with continuous variables and expressed as mean ± standard deviation or median with interquartile range. We used paired t-tests when applicable to compare continuous variables and chi-square tests for categorical data. Since there are no existing randomized study on whether SGLT2i administration improves diastolic function, except for animal studies, the number of subjects was calculated as follows with reference to previous studies that observed changes in diastolic function (E/e’) [12, 13]. A sample size of approximately 99 was determined to be necessary to detect medium effect size of difference in E/e’ with 80% power, allowing approximately a 10% dropout rate. The clinically meaningful difference chosen for this sample size calculation was based on observations that mitral annular relaxation velocity from diabetes and nondiabetes populations differed by approximately 2.2 [2]. All analysis was conducted with IBM SPSS ver. 27.0 (IBM Corp). Statistical significance was concluded at a two-sided significance level of 0.05 for all analyses.

## Results

**Fig. 1:**
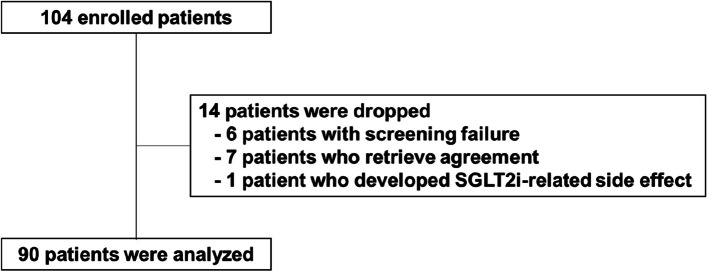
Study flowchart**.** SGLT2i, Sodium-glucose cotransporter type 2 inhibitor

Among 104 patients who were enrolled in this study, 14 patients were excluded from final analysis due to withdrawal of their consent or medication-related adverse events (Fig. 1). Baseline characteristics are shown in Table 1. Mean age was 56 years old and 76.6% was male. Most patients (86.7%) had three or more cardiovascular risk factors, and 29 patients (32.2%) had all five risk factors as well as diabetes. Before administration SGLT2i, patients took single or combination agent for glucose control including metformin, dipeptidyl peptidase-4 inhibitor, sulfonylurea, thiazolidinedione, or insulin. There was significant improvement in parameters reflecting glucose control status after administration of SGLT2i (Table 2). HbA1c levels were significantly lowered from 8.08% ± 1.09% to 7.31% ± 1.12% (*P* < 0.001). However, the level of NT-proBNP was within normal range at the time of enrollment (44.5 ± 39.8 pg/mL) and did not differ after the medication of SGLT2i (42.2 ± 40.9 pg/mL, *P* = 0.745). Since the majority of enrolled patients were under the medication of lipid-lowering agent, the lipid profile was in well-controlled status. There were no adverse events or mortalities until the end of follow-up period leading up to May of 2024.

**Table 1:** Baseline characteristics

Characteristic	Value (*n* = 90)
Age (yr)	56.0 ± 10.3
Hypertension	66 (73.3)
Well-controlled (BP < 140/90 mmHg)	39 (43.3)
Uncontrolled (BP ≥ 140/90 mmHg)	27 (30.0)
Smoking	57 (63.3)
Ex-smoker	30 (33.3)
Current smoker	27 (30.0)
Insulin	20 (22.2)
Dyslipidemia	79 (87.8)
Obesity (BMI ≥ 30 kg/m^2^)	73 (81.1)
No. of risk factors	
≥ 3	78 (86.7)
≥ 4	58 (64.4)
≥ 5	29 (32.2)
Medication	
Calcium channel blocker	21 (23.3)
β-blocker	3 (3.33)
ARB/ACEi	59 (43.3)
Statin	68 (75.6)
Antithrombotic agent	27 (30.0)
Dipeptidyl peptidase-4 inhibitor	64 (71.1)
Metformin	86 (95.5)
Sulfonylurea	36 (40.0)
Thiazolidinedione	1 (1.11)
Insulin	20 (22.2)

Values are presented as mean ± standard deviation or number (%)

*BP* blood pressure, *BMI* body mass index, *ARB* angiotensin receptor blocker, *ACEi* angiotensin converting enzyme inhibitor

**Table 2:** Changes of laboratory findings

Initial laboratory finding	Pre-SGLT2i	Post-SGLT2i	Difference	*P*-value
White blood cell count (10^3^/μL)	7.45 ± 1.50	7.35 ± 1.44	0.12 ± 1.23	0.354
Hemoglobin (g/dL)	14.4 ± 1.36	15.0 ± 1.41	–0.58 ± 0.72	< 0.001
Platelet count (10^3^/μL)	232.3 ± 57.5	232.2 ± 62.9	0.75 ± 23.5	0.767
Protein (g/dL)	7.22 ± 0.46	7.41 ± 0.49	–0.20 ± 0.38	0.004
Albumin (g/dL)	4.55 ± 0.35	4.51 ± 0.32	0.03 ± 0.27	0.486
Blood urea nitrogen (mg/dL)	14.7 ± 4.58	16.7 ± 4.65	–2.02 ± 3.58	< 0.001
Creatinine (mg/dL)	0.85 ± 0.19	0.86 ± 0.20	–0.01 ± 0.10	0.603
Glomerular filtration rate (mL/min/1.73m^2^)	92.9 ± 13.9	91.5 ± 17.0	1.10 ± 10.4	0.324
Total cholesterol (mg/dL)	146.0 ± 36.7	144.2 ± 37.1	1.64 ± 23.0	0.503
Trigliceride (mg/dL)	183.5 ± 173.8	169.6 ± 210.6	7.91 ± 105.7	0.482
High-density lipoprotein (mg/dL)	46.2 ± 9.90	47.4 ± 10.9	–0.94 ± 6.51	0.174
Low-density lipoprotein (mg/dL)	84.1 ± 32.7	80.1 ± 26.1	4.20 ± 21.1	0.063
Fasting glucose (mg/dL)	163.9 ± 35.9	134.3 ± 29.4	28.8 ± 38.6	< 0.001
Hemoglobin A1C (%)	8.08 ± 1.09	7.31 ± 1.12	0.76 ± 0.73	< 0.001
Glycoalbumin (%)	20.2 ± 4.55	18.5 ± 4.47	2.48 ± 2.89	< 0.001
NT-proBNP (pg/mL)	44.5 ± 39.8	42.2 ± 40.9	1.82 ± 32.8	0.745
Body mass index (kg/m^2^)	28.2 ± 4.0	27.3 ± 4.0	–0.9 ± 0.9	< 0.001

Values are presented as mean ± standard deviation

*SGLT2i* sodium-glucose cotransporter type 2 inhibitor, *NT-proBNP* N-terminal pro-brain natriuretic peptide

**Fig. 2:**
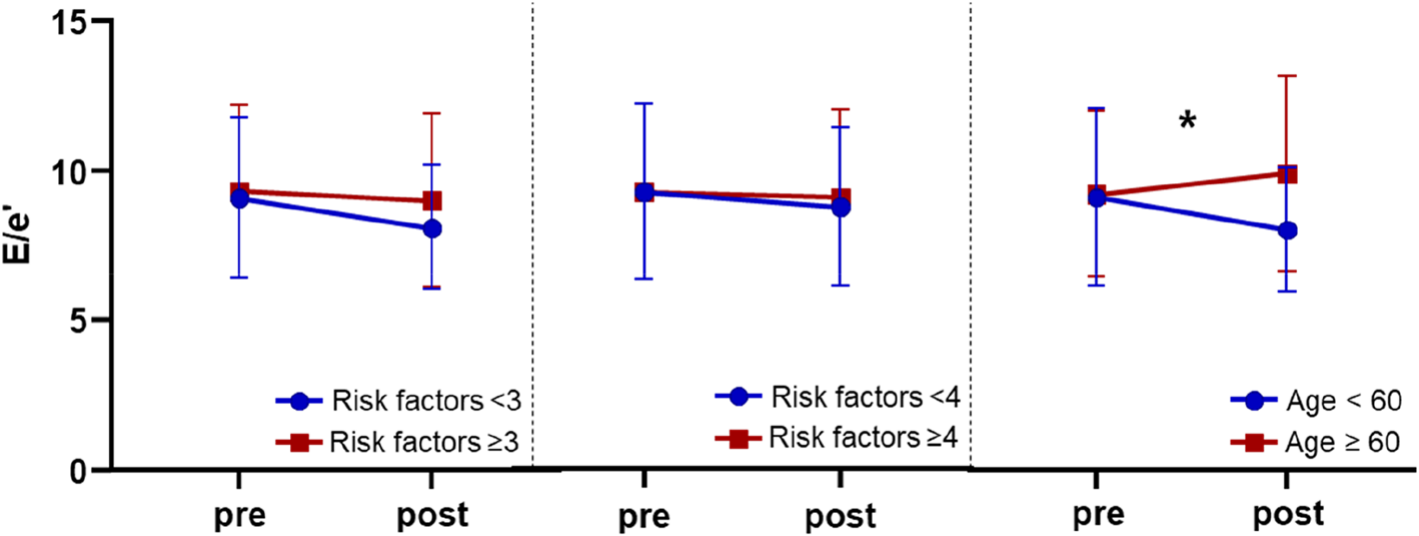
Changes of E/e’ according to the presence of cardiovascular risk factors

Table 3 presents changes of echocardiographic findings. At baseline, cardiac systolic function (LV ejection fraction [LVEF], 64.3% ± 5.59%) and diastolic function (E/e’, 9.23 ± 2.89; LAVI, 28.0 ± 8.20 mL/m^2^) were within normal range, as these patients had no documented history of HF or cardiomyopathy. Although the decrease in E/e’ after the administration of SGLT2i was observed in 18 patients (20.0%), there was no significant difference in average E/e’ value (*P* = 0.235) or LAVI (*P* = 0.936) before and after the medication. Regardless of the number of cardiovascular disease-complicating risk factors, E/e’ value remained unchanged before and after the SGLT2i treatment (Fig. 2). Even in patients with all known risk factors including old age, E/e’ value did not decrease after adding SGLT2i (8.9 ± 2.4 vs. 8.7 ± 3.2).

**Table 3:** Changes of echocardiographic parameter

Echocardiographic finding	Before medication	After medication	Difference	*P*-value
LV end-diastolic dimension (mm)	49.0 ± 3.89	47.9 ± 3.89	1.02 ± 4.14	0.021
LV end-systolic dimension (mm)	29.1 ± 3.22	30.4 ± 20.9	–1.34 ± 21.7	0.558
LVEF (%)	64.3 ± 5.59	71.2 ± 56.2	–6.83 ± 57.1	0.259
Interventricular septal thickness (mm)	9.24 ± 1.12	9.07 ± 1.29	0.18 ± 1.17	0.16
Mitral inflow E velocity (m/sec)	0.62 ± 0.15	0.61 ± 0.13	0.01 ± 0.15	0.534
Mitral inflow A velocity (m/sec)	0.72 ± 0.15	0.72 ± 0.15	0.00 ± 0.15	0.863
E/A ratio	0.88 ± 0.24	0.88 ± 0.28	–0.00 ± 0.26	0.916
Deceleration time (msec)	233.6 ± 45.2	233.1 ± 51.6	0.50 ± 59.3	0.936
Septal e' velocity (m/sec)	0.07 ± 0.02	0.07 ± 0.02	–0.00 ± 0.02	0.117
A' velocity (m/sec)	0.10 ± 0.02	0.10 ± 0.02	0.00 ± 0.02	0.797
E/e'	9.23 ± 2.89	8.89 ± 2.81	0.34 ± 2.73	0.235
LAVI (mL/m^2^)	28.0 ± 8.20	27.8 ± 6.78	0.06 ± 7.60	0.936
Difference of E/e’ ≥ 2.2	-	-	18 (20.0)	-

Values are mean ± standard deviation

*LV* left ventricular, *LVEF* left ventricular ejection fraction, *LAVI* left atrial volume index

There were no significant differences in baseline characteristics between the two age groups. However, there was a statistical difference in E/e’ change after the SGLT2i administration between patients younger than 60 years and those older than 60 years (–0.7 ± 2.2 vs. 1.1 ± 2.8, *P* = 0.002). While there was no difference of E/e’ value in patients older than 60 years, there was a significant decrement of E/e’ in those younger than 60 years after the medication. Although not statistically significant due to the small number of patients, those under 60 years tended to show greater reductions in both body mass index (–0.99 ± 0.96 kg/m^2^ vs. –0.79 ± 0.70 kg/m^2^) and LV mass index (–3.96 ± 15.2 g/m^2^ vs. –1.22 ± 14.80 g/m^2^) compared to those over 60 years after SGLT2i administration.

## Discussion

In the present study we evaluated the change of LV diastolic function in patients with T2DM who started SGLT2i. Despite the fact that most patients had potential risk factors for cardiovascular complication, there were no significant changes of LV diastolic parameters after SGLT2i administration. Even in the high-risk group for diabetes-related cardiovascular events, the diastolic profile did not improve after additional SGLT2i administration. In patients younger than 60 years of age, E/e' was significantly decreased after administration of SGLT2i compared to patients aged 60 years or older.

Currently, SGLT2i is strongly recommended for T2DM patients with established ASCVD, HF, or chronic kidney disease [10, 11]. In addition, newly updated HF guidelines also recommend SGLT2i for T2DM patients with either established cardiovascular disease or high risk for it in order to prevent hospitalization for HF [15–17]. However, the evidence are weak as to whether SGLT2i should be considered as a primary antidiabetic agent in patients who have only indicators of high risk for cardiovascular complication without established HF or other types of cardiovascular disease. Recent clinical trials consistently showed that SGLT2i improved the rates of HF hospitalization in patients with T2DM, but most of enrolled patients already had ASCVD [5, 6, 18]. In patients without documented cardiovascular disease, benefit of SGLT2i over other types of antidiabetic agents in terms of glucocentric and cardiovascular outcome is unclear.

Since LV diastolic dysfunction is considered as the early clinical manifestation of diabetic cardiomyopathy including HF with reduced ejection fraction as well as HF with preserved ejection fraction, several trials have assessed the improvement of diastolic parameters after the administration of SGLT2i in patients with T2DM. A meta-analysis on 11 randomized controlled trials showed a trend to decrease cardiac volume indices and improve of functional parameters in patients using SGLT2i, which were not statistically significant [19]. Soga et al. [12] prospectively showed that the E/e' significantly decreased 6 months after the administration of dapagliflozin in 57 diabetic patients. However, all patients enrolled in the study were under stable HF. Matsutani et al. [13] also reported improvement of E/e’ after 3 months treatment with canagliflozin in 37 diabetic patients, but about a third of enrolled patients had preexisting cardiovascular disease. Meanwhile, a recent randomized controlled trial in Korea demonstrated that SGLT2i did not significantly affect resting e’ velocity, E/e’, LAVI despite it improved diastolic parameter after exercise [14]. Taken together, the effect of SGLT2i on diastolic function is inconclusive for the following reasons: (1) enrolled patients mostly had documented cardiovascular disease; (2) the treatment duration was heterogeneous; and (3) sample sizes were too small to draw clear conclusion.

In our data, initial LV diastolic function was mostly within the normal range despite potential risk factors of enrolled patients for cardiovascular disease. Nevertheless, since E/e’ value linearly correlates with LV filling pressure, its decrement is meaningful for indicating diastolic function improvement [20, 21]. However, there was no significant decrease in E/e’ value 3 months after administration of SGLT2i in our study. A recent cohort study demonstrated that first-line diabetic treatment with SGLT2i had similar effect for cardiovascular outcome and safety profiles compared to metformin [22]. These results might support that most apply a glucocentric approach and recommend other types initial medication such as metformin for most diabetics without cardiovascular disease, leaving SGLT2i as an alternative option.

Generally, LV diastolic dysfunction is highly prevalent in elderly patients whose relaxation of myocardium is impaired with increasing age. Our data showed that the change of E/e’ was significantly different between patients younger and older than 60 years. In our data, patients under 60 years tended to show greater reductions in body mass index and LV mass index after SGLT2i treatment, which may have contributed to the observed improvement in E/e', potentially due to greater myocardial reserve enhancing their response to the cardioprotective effects of SGLT2i, although these trends were not statistically significant due to the small sample size. Considering that SGLT2i induces volume depletion, blood pressure reduction and weight reduction especially in elderly patients, a patient-centered choice of antidiabetic drugs for balancing of benefits and harms across patients with T2DM with different cardiovascular and/or kidney risk is needed.

There are some limitations of the current study. First, the study was designated as a single-arm prospective observation, so we could not compare the effectiveness of SGLT2i on cardiac function with patients who took other types of glucose-lowing agents. Second, this study comprised a small number of patients, but our data enrolled relatively the largest number of patients compared with previous studies dealing with the benefit of SGLT2i on diastolic function, and which met the sample size calculation based on the significance of E/e’ change. Third, the present study was focused on the effect of SGLT2i on changes of diastolic parameter as early markers of diabetic cardiomyopathy in patients without cardiovascular disease despite high potential risk factors. Lastly, the relatively short duration of SGLT2i administration may have contributed to the lack of significant changes in diastolic function. However, previous studies that observed the effects of SGLT2i on diastolic function in diabetic patients similarly assessed changes after 3 to 6 months of treatment, based on preclinical research supporting the cardioprotective effects of SGLT2i. Further observation studies are warranted to evaluate the impact of long-term SGLT2i therapy on cardiac function in the context of diabetic cardiomyopathy.

Therefore, baseline E/e’ value was not elevated, so it could be limited to detect changes of diastolic parameter by administration of SGLT2i. However, considering that the E/e’ value closely correlates with LV filling pressure and pulmonary capillary wedge pressure in HF with or without decrement of LVEF, we thought that the change of E/e’ within normal range could be meaningful. Lastly, the relatively short duration of SGLT2i administration may have contributed to the lack of significant changes in diastolic function. However, previous studies that observed the effects of SGLT2i on diastolic function in diabetic patients similarly assessed changes after 3 to 6 months of treatment, based on preclinical research supporting the cardioprotective effects of SGLT2i. Further observation studies are warranted to evaluate the impact of long-term SGLT2i therapy on cardiac function in the contexed of diabetic cardiomyopathy.

## Conclusions

In T2DM patients without documented cardiovascular disease including HF, administration of SGLT2i showed no improvement in diastolic function profile. It is necessary to reconsider SGLT2i as a priority in the selection of antidiabetic drugs in patients that are at potential risk for cardiovascular disease. Further larger-scale randomized studies are needed to determine which patients will benefit from potential cardiovascular events with early addition of SGLT2i.

## Data Availability

No datasets were generated or analysed during the current study.

## Abbreviations

ASCVD: Atherosclerotic cardiovascular disease
HbA1c: Hemoglobin A1C
HF: Heart failure
HOMA-IR: Homeostatic model assessment for insulin resistance
LAVI: Left atrial volume index
LV: Left ventricular
LVEF: Left ventricular ejection fraction
NT-proBNP: N-terminal pro-brain natriuretic peptide
SGLT2i: Sodium-glucose cotransporter type 2 inhibitor
T2DM: Type 2 diabetic mellitus
